# One month follow up of a neonate born to a mother who survived Ebola virus disease during pregnancy: a case report in the Democratic Republic of Congo

**DOI:** 10.1186/s12887-019-1584-6

**Published:** 2019-06-18

**Authors:** N. Kasereka Baraka, Mupenzi Mumbere, Evie Ndombe

**Affiliations:** 1 Department of Paediatrics, Faculty of Medicine, University of Kalemie, Kalemie, Democratic Republic of the Congo; 2 Department of Paediatrics, Faculty of Medicine, Catholic University of Graben, Butembo, Democratic Republic of the Congo; 3 Department of Obstetrics and Gynaecology, Faculty of Medicine, University of Kalemie, Kalemie, Democratic Republic of the Congo

**Keywords:** Case report, Ebola virus, Neonate, Pregnancy, One month follow up

## Abstract

**Background:**

The authors report a 1 month follow up of a neonate described as “miracle baby” because she was born Ebola virus disease-free and survived after her mother was infected with Ebola virus during the third trimester of pregnancy.

**Case presentation:**

This female newborn baby was registered at the Maternity of Beni Reference General Hospital and the Ebola Treatment Centre in eastern Democratic Republic of Congo. She was delivered normally and showed no signs of Ebola infection. All tests were negative for Ebola. At 1 month follow up, the baby is growing normally.

**Conclusions:**

This very rare happy outcome for neonates of mothers infected with Ebola virus motivated the authors to report the case.

## Background

According to the World Health Organization (WHO), Ebola virus disease (EVD) is a serious, highly contagious and fatal disease in humans. The virus is transmitted to humans from wild animals and spreads to the population through interpersonal transmission [1].

Ebola virus disease during pregnancy is associated with a very high rate of obstetric complications and perinatal adverse outcomes including spontaneous abortion, premature rupture of membranes, premature delivery, ante- and postpartum haemorrhage, intrauterine death of the foetus, maternal and neonatal death [2]. Neonates born to women with Ebola virus (EBOV) are often premature [3] and typically do not survive for more than a few weeks [4].

The data show that intrauterine content remains positive for Ebola virus ribonucleic acid (RNA) [5]. Since the identification of EBOV, 15 neonates born alive to EBOV-infected mothers have been documented. All died; the longest documented survival was 19 days [6].

During the 2014–2016 outbreak, Médecins Sans Frontières treated at least 54 EBOV-infected women with pregnancies in the second and third trimester and recorded 35 second-trimester miscarriages and deliveries in Ebola treatment centres. The single baby born alive died 2 days after birth. To date, there is only one survival beyond neonatal period reported in the literature about babies born to EBOV-infected women. It was a female baby born with congenital EVD who was followed up for 12 months of life in Conakry (Guinea) and was found to be growing normally [7]. While in a retrospective cohort study of patients managed at 5 Ebola Treatment Units (ETU) in West Africa, two neonates born live to EBOV-infected women in the ETU died within 8 days [8].

Here we report a 1 month follow up of a neonate born to a mother who survived the Ebola virus disease in eastern Democratic Republic of Congo during the Ebola outbreak that was declared in August 2018 and continues to wreak havoc in the northern areas of the province of Nord-Kivu and the neighbouring areas in the province of Ituri.

## Case presentation

Our patient’s mother, 22-year-old previously healthy multigravida woman (Para 4, Gravida 6, Abortion 1, Live children 4) was admitted to the Beni General Hospital Ebola Treatment Center 2 days after onset of symptoms (fever, vomiting and malaise) and was confirmed positive for EBOV by polymerase chain reaction (PCR). She reported a 34 week pregnancy and confirmed that the previous pregnancy follow up was uneventful. She was discharged after 1 week of management made of rehydration, Cefixime, Paracétamol and other supportive measures.

At the gestational age of 38 weeks, vaginal delivery occurred after a 10 h labor, the foetus was in breech presentation with meconial amniotic fluid and a normal placenta. The female newborn baby did well initially with an Apgar score of 8/9/10. General and neurologic examination did not reveal any pathology. The vital signs and anthropometric parameters at birth were as follows: temperature 36.5 °C, regular breathing at 52 cycles per minute and heart rate 146 beats per minute; weight of 3500 g, head circumference of 36 cm and height of 53 cm. The haemoglobin level was 16 g / dl; the umbilical cord blood PCR, the blood and salivary swabs were negative for the Ebola virus disease. She was subjected to an antibiotic therapy made of cefotaxime (3x200mg/day) and was discharged after 5 days.

At 1 month and 6 days of age, the baby was growing normally, she weighed 4100 g (versus 4400 g ideally), she was being breastfed and her mother reported no illness in the past days of her life.

## Discussion and conclusions

We describe a neonate who was born EBOV-free from a mother who survived Ebola virus disease during her pregnancy in late stage. We think that this favourable outcome was probably due to the fact that the mother presented a relatively mild disease phenotype which was not transmitted to the foetus in utero.

Although information on the consequences of Ebola during pregnancy is limited (in terms of risk factors), early reports seemed to indicate very high mortality, which also leads to a fatalistic approach to the pregnant population management [5, 9]. However in a retrospective cohort study of patients managed at five Ebola treatment units in West Africa, the data do not support the idea that pregnant women are at higher risk for death compared with nonpregnant patients with EVD [8].

According to very disturbing reports from West Africa, pregnant women suspected of having Ebola virus were denied access to health facilities because healthcare providers feared they would infect other patients. Because of the history of an almost uniformly fatal outcome in infants born to mothers with Ebola, these pregnant mothers and their infants have not been given the care needed [10]. However throughout the course of the epidemic, the situation improved over time [8].

Although these are very rare cases, some pregnant women have survived the Ebola virus disease without losing the unborn child. In a medical facility in developed countries, the chances of survival of a pregnant woman with Ebola virus disease could be high thanks to the implementation of standard supportive care. However, whether supportive care for neonates born to women infected with EVD would be effective in high income settings also remains to be seen [10].

Ebola virus disease remains a serious public health problem because of its high mortality and the fatal obstetrical and neonatal complications. Our case highlights the possibility of delivery and survival of disease-free full term babies born to mothers who survived the Ebola virus disease. Thus, care should be strengthened in pregnant women to improve the chances of saving neonates born in these conditions.

## Data Availability

The datasets used and/or analysed during the current study are available from the corresponding author on reasonable request.

## Abbreviations

EBOV: Ebola virus
ETU: Ebola treatment unit
EVD: Ebola virus disease
PCR: Polymerase chain reaction
RNA: Ribonucleic acid
WHO: World Health Organization

## References

[1] OMS (2014). Prévention et contrôle de l’infection pour les soins aux cas suspects ou confirmés de fièvre hémorragique à filovirus dans les établissements de santé, avec un accent particulier sur le virus Ebola.

[2] Baggi F M, Taybi A, Kurth A, Van Herp M, Di Caro A, Wölfel R, Günther S, Decroo T, Declerck H, Jonckheere S. Management of pregnant women infected with Ebola virus in a treatment centre in Guinea, June 2014. Euro Surveill 2014;19(49):pii=20983. 10.2807/1560-7917.ES2014.19.49.20983. 10.2807/1560-7917.es2014.19.49.20983 25523968

[3] Wamala JF, Lukwago L, Malimbo M, Nguku P, Yoti Z, Musenero M (2010). Ebola hemorrhagic fever associated with novel virus strain, Uganda 20O7-2008. Emerging Infect Dis..

[4] Francesconi P, Yoti Z, Declich S, Onek PA, Fabiani M, Olango J (2003). Ebola hemorrhagic fever transmission and risk factors of contacts, Uganda. Emerging Infect Dis.

[5] Jameison DJ, Uyeki TM, Coologhan WM, Meaney D, Rasmussen SA (2014). What obstetrician-gynecologist should know about Ebola; a perspective from the center for disease control and prevention. Obstest Gynecol.

[6] Nelson JM, Griese SE, Goodman AB, Peacock G (2015). Live neonates born to mothers with virus disease: a review of the literature. J Perinatol.

[7] Dornemann J, Burzio C, Ronsse A, Sprecher A, De Clerck H, Van Herp M (2017). First newborn baby to receive experimental therapies survives Ebola virus disease. J Infect Dis.

[8] Henwood PC, Bebell LM, Roshania R (2017). Ebola virus disease and pregnancy: a retrospective cohort study of patients managed at 5 Ebola treatment units in West Africa. Clin Infect Dis.

[9] Mupapa K, Mukundu W, Bwaka MA, Kipasa M, De Roo A, Kavula K (2000). Ebola hemorrhagic fever and pregancy. J Infect Dis.

[10] Lang, J. Ebola in the maternity ward. New York. 2014. http://www.newyorker.com/com/tech/elements/ebola-ward. Accessed 02 Feb 2019.

